# Cutting Through Barriers: Laceration Showcase and Virtual Procedure Lab for Emergency Medicine Trainees in India

**DOI:** 10.7759/cureus.93007

**Published:** 2025-09-23

**Authors:** Susan Owens, Uche Anigbogu, Amy Kiem, Katherine Douglass, Tania Ahluwalia

**Affiliations:** 1 Emergency Medicine, University of Kentucky, Lexington, USA; 2 Emergency Medicine, George Washington University, Washington, D.C., USA; 3 Pediatric Emergency Medicine, Children's National Hospital, Washington, D.C., USA

**Keywords:** emergency medicine, emergency medicine resident, global health trainee, laceration, procedure training

## Abstract

Procedural training for Emergency Medicine (EM) trainees is a critical component of physician development. Building on previous virtual pilot projects, this project aimed to assess the feasibility and engagement of a remote procedural training program for Indian postgraduate EM trainees.The primary goal of this project was to design and implement a competitive remote procedural skill showcase followed by a virtual procedure lab for advanced skill development. To further enhance the learner experience and foster intrinsic motivation, gamification strategies were leveraged through a visible leaderboard and peer-voted recognition, providing both formative feedback and peer-based validation.

Participants submitted photographs of laceration repairs that were evaluated by two blinded reviewers. The top scorers presented to their peers, and a winner was chosen by peer vote. The showcase was followed by a virtual suture workshop led by a wound care expert and supported by multiple facilitators, allowing large group demonstration and individual practice with directed feedback in small breakout rooms.51 participants from PGY1-3 levels submitted laceration repairs; each PGY class chose a winner, with secondary awards for most dramatic laceration repair and best pediatric laceration repair. This was followed by an interactive virtual suture lab to practice advanced repair techniques. 160 trainees attended the virtual suture lab.

This technical report highlights the project’s feasibility in delivering engaging training in a remote environment. The combination of structured evaluation, blinded review, and facilitated procedure practice with individualized feedback likely increased engagement compared to the previous pilot project. The project received informal positive feedback and demonstrated the feasibility and acceptability of remote procedural teaching, paving the way for future scalable and cost-effective models.

## Introduction

Procedural training is an essential component of emergency medicine (EM) training. In the context of India’s growing EM programs, skill development in clinical procedures remains a challenge due to logistical and resource constraints. Traditional hands-on procedural training models often rely on in-person sessions, which can be costly and inaccessible for trainees across geographically diverse and underserved areas. For virtual medical education programs, maintaining learner engagement and the integrity of procedural training are two consistent difficulties that are affected by time zone differences, internet access and reliability, and materials availability. This project aimed to bridge these gaps by providing an engaging structured remote platform for procedural skill development for laceration repair.

In addition to EM training program requirements, laceration repair is a critical skill in India due to the high volume of minor and major traumas and significant morbidity associated with wound complications, including cosmesis and infection [1,2]. This project specifically sought to improve laceration repairs for trainees in postgraduate EM (PGY1-3), ensuring scalability, inclusivity, and quality education tailored to local needs. The paired competitive laceration showcase and virtual suture lab is the second iteration of a pilot project started during the COVID-19 pandemic to maintain procedural training in resource-variable environments [3]. 

The Ronald Reagan Institute of Emergency Medicine (RRIEM) at the George Washington University (GWU) has collaborated with partners in India since 2006 in 3-year post-graduate EM training programs called the Masters in Emergency Medicine (MEM) program, currently supporting 22 sites [4,5]. This is an institution-led partnership that has contributed significantly to the development of EM and system strengthening in India. Training includes didactics, clinical rotations, research, and annual examinations executed using in-person faculty education and thrice-weekly online video conferencing and webinars. 

## Technical report

The primary objective of this project was to design and implement an engaging competitive remote procedural skill showcase for EM trainees in India, followed by a virtual procedure lab for advanced skill development. This project builds on the previously described virtual procedure lab for basic laceration repair techniques that was implemented in 2022 [3]. In the previous pilot project, only 21 trainees participated in the suture skills project outside of the virtual procedure lab that was attended by 160 trainees (56% of total enrollees) [3]. This project was designed to boost learner engagement with the workshop by generating a competitive environment to showcase the baseline learner skill and variety of clinical cases managed by MEM program trainees.

The 2022 pilot remote procedural training model for basic laceration repair was developed based on adult learning principles and Peyton’s stages of skill acquisition [3,6]. The same methodology was used in this iteration of the virtual procedure lab with specific improvements: focus on advanced suture techniques (corner stitch, vertical and horizontal mattress, and flap repair) based on prior trainee feedback and increase the learner-to-instructor ratio by recruiting a broader faculty base (EM faculty, EM fellows, advanced practice providers) which allowed for breakout rooms for individualized real-time feedback and skill refinement during the virtual lab.

To further enhance the learner experience and foster intrinsic motivation, we leveraged gamification strategies through a visible leaderboard and peer-voted recognition, providing both formative feedback and peer-based validation [7,8]. Rubric development was central to the project, ensuring a structured evaluation of submissions, and was based on the technical components of a laceration repair procedure note, including wound shape and size, presence of foreign bodies or contamination, suture type, and suture number. Participants were required to submit photographs of their laceration repairs after obtaining verbal consent, which two blinded reviewers subsequently evaluated to maintain fairness. Characteristics of the submissions are presented in Table 1. The reviewers were core faculty for the GWU-MEM program who are practicing EM physicians, well-versed in laceration repair and documentation. In instances of discrepancy between the two reviewers, a third reviewer scored the submissions in question.

**Table 1:** Characteristics of Laceration Showcase Submissions

Characteristic	Category	Value
Sites Represented		15
PGY year	PGY-3	12
	PGY-2	15
	PGY-1	24
Pediatric Submissions		5
Laceration Location	Distal extremity (excluding fingers/toes)	9
	Hand, Fingers or Toes	5
	Proximal extremity, Trunk or Chest	4
	Face or Scalp	30
	Neck	3
Laceration size	1-3 cm	9
	4-6 cm	20
	7-9 cm	9
	10-12 cm	5
	>12 cm	8
Complexity of Repair	Single layer of simple interrupted sutures	35
	Presence of mattress sutures	6
	Multi-layer repair	10

All EM trainees from partner sites across India were notified of the laceration showcase competition and virtual procedure lab via weekly programmatic emails and reminded during virtual teaching sessions on Zoom. Participants were provided with laceration showcase submission instructions six weeks in advance of the submission deadline; trainees were instructed to submit a before-and-after photo of a laceration repair performed by the trainee in the Emergency Department with a short case summary. The scoring process utilized a detailed rubric based on the technical components of a laceration repair, including assessment of size, location, depth, presence of contamination, and suture technique. The project emphasized objectivity and consistency by engaging two blinded evaluators per submission.

Significant score differences that would lead to a change in ranking that would effect submissions for the large group showcase were settled by engaging a third evaluator. All evaluators were practiticing EM physicians who regularly engage in resident education. The top five scores from each PGY class were selected for a showcase presentation for their peers. After the presentations, trainees voted in real-time using a live Zoom poll, selecting their favorite laceration repairs, and winners were announced one week later at a ceremony preceding the virtual procedure lab. In preparation for the virtual procedure lab, notification was provided to trainees and site directors four weeks in advance.

Recognizing the variability in material access across India, equitable participation was emphasized by recommending locally available substitutes such as raw chicken breast, fruit with a peel, and foam block covered in fabric for simulation materials, thus improving access and inclusivity. The virtual suture workshop occurred during the weekly grand rounds which was hosted on Zoom by a wound care expert and supported by multiple facilitators, allowing large group demonstration and individual practice with directed feedback in small breakout rooms.

## Discussion

Prior to the COVID-19 pandemic, this activity would have been delivered at individual sites by visiting faculty. Using the PICRAT framework - a technology integration in education framework, where PIC stands for passive, interactive, or creative, and RAT stands for replaces, amplifies, or transforms, discussed at length in Khamees et al’s systematic review of remote learning developments since the pandemic - this education development aligns with a replacement strategy through the use of technology that is interactive for the learner [9]. To further increase learner engagement and participation with the procedure lab, a pre-lab laceration showcase was added to this iteration of the wound management curriculum, which achieved a high level of engagement, with 51 trainees submitting photo demonstrations. While the voluntary nature of the pre-workshop laceration showcase is based on the self-determination theory of gamification, voluntary submission also introduces selection bias [10]. The skills demonstrated in the showcase submissions may not represent the true skill variability among trainees, and the self-motivation among trainees may not be consistent over time, affecting the reproducibility of this perceived success. 

The characteristics of the 51 submissions are shown in Table 1. Three submissions were repaired in the operating theater and were removed from the general score rankings but acknowledged during the awards ceremony for impressive mechanisms. The most common laceration location was the face or scalp (n = 30), followed by the distal extremity (n = 9). PGY-1s primarily used the simple interrupted technique (n = 18 of 24 PGY-1 submissions) while PGY-2/3 submissions included several layered closures and complex facial lacerations. These characteristics underscore the decision to focus this iteration of the virtual suture workshop on advanced suture techniques, including corner stitch, vertical and horizontal mattress, and flap repairs. 

Scores were noted to be higher for PGY-2 and PGY-3 submissions compared to PGY-1 submissions, which was expected, though the scoring process revealed variability in procedural proficiency across trainees and concerns from evaluators regarding repair decisions, such as technique in relation to location and suture material, demonstrating the importance of sustained training and mentorship. Impressed with the variety of submissions, additional recognition was given to top-scoring pediatric laceration repairs for each class. While the initial showcase submissions were objectively evaluated based on common components of laceration repair documentation, peer-voting for showcase winners may have introduced bias that could affect the reproducibility of this intervention. 

This project capitalizes on several best practices recommended by the Accreditation Council for Graduate Medical Education (ACGME) for planning effective remote synchronous didactics including use of a consistent platform, set ground rules for participation including naming convention and active use of video, use of gamification to capture learner interest, and use a combination of large group and small group formats to increase engagement [11]. The degree of participation in the challenge and informal discussion with trainees indicated great enthusiasm that positively influenced participation in the virtual procedure lab, which 160 trainees attended (of 264 total enrollees, n=61%). 

Participants expressed enthusiasm for the training, with written and verbal feedback during the suture workshop indicating that the structured format and detailed evaluations were well-received. Given the positive response to the 2022 pilot and similar informal feedback for this iteration of the procedure lab, we did not solicit formal feedback. The results demonstrated the feasibility of implementing a remote procedural training program and its potential to fill critical educational gaps in resource-limited settings. Additionally, the greater participation in a pre-workshop showcase (n=51 in the 2025 pre-workshop showcase versus n = 21 in the 2022 post-workshop challenge) potentially contributed to increased participation in the suture workshop (61% of enrollees in 2025 versus 57% of enrollees in 2022), although causation would be difficult to determine. 

The GWU-MEM program operated remotely during the COVID-19 pandemic and continues to operate in a hybrid design; thus, learners are familiar with the Zoom platform. Ongoing difficulties with internet connection are common and often result in interruptions, but learners can rejoin and turn off video to preserve bandwidth, which speaks to the resiliency of this group of learners. This project highlights the potential of remote procedural training to address challenges in skill development among EM trainees across resource-variable settings.

This was a two-part virtual suture skills innovation combining a live hands-on procedure lab with real-time feedback and an independent competitive skill demonstration that engaged 51 trainees who voluntarily submitted their work for critical appraisal and 160 trainees in a virtual procedural workshop. The project received informal positive feedback and demonstrated the feasibility and acceptability of remote procedural teaching, paving the way for future scalable and cost-effective models. The combination of structured evaluation, blinded review, and facilitated procedure practice with individualized feedback was key to the project’s success. The opportunity to highlight clinical cases managed by trainees bolstered interest and participation in virtual sessions, as well as contributed to increased efforts for bi-directional knowledge sharing across the greater RRIEM program.

We noted that different materials were used in the submissions compared to practice in the United States; for example, fast gut is often used in pediatric lacerations as it is an absorbable suture, which was not available at all the sites and was not used in any of the submissions. The study also revealed limitations, including the reliance on participant-provided photographs without video documentation, which likely contributed to score differences amongst evaluators. The ease of photo submission compared to video submission may have contributed to greater participation in the extra-workshop activity; similar difficulties with learner video submission have been noted in other similarly designed virtual suture workshops [12]. Despite these challenges, the project provides a valuable proof of concept for leveraging virtual platforms to deliver high-quality, cost-effective procedural training.

## Conclusions

Our findings suggest that this dual-modality model, a competitive asynchronous component followed by a synchronous faculty-led lab, provides an acceptable model amongst trainees for procedural training in low-resource settings and could be expanded to other procedural skills in resource-variable settings. Use of a competitive pre-workshop activity capitalizes on current gamification trends in medical education, with ease of pre-workshop activity directions contributing to increased learner participation. Future iterations of the project will focus on expanding participant cohorts, refining evaluation tools, incorporating pre- and post-training assessments to evaluate long-term impacts on clinical competency, and involving more local facilitators to enhance scalability and sustainability.

Additionally, a third iteration combining a pre-workshop engagement activity and a post-workshop skill demonstration activity with a virtual procedure workshop that incorporates pre-and post-training assessments may bolster skill retention and achieve higher levels in the Kirkpatrick Learning Model. This work contributes to the growing body of work on virtual procedure workshops and provides another modicum to improve learner engagement.

## References

[1] Harna B, Arya S, Bahl A (2020). Epidemiology of trauma patients admitted to a trauma center in New Delhi, India. Indian J Crit Care Med.

[2] Dany James J, Sharma SL, Agrawal D (2025). Trauma in India: current status and the path forward. Trauma Surg Acute Care Open.

[3] Owens S, Ahluwalia T, Douglass K, Gidwani S (2022). Sustaining capacity building and practical skills training during the COVID-19 pandemic: lessons from India. AEM Educ Train.

[4] Douglass K, Pousson A, Gidwani S, Smith J (2015). Postgraduate emergency medicine training in India: an educational partnership with the private sector. J Emerg Med.

[5] (2025). Partner institutions - the Ronald Reagan Institute. https://smhs.gwu.edu/reaganinstitute/international/india/partners.

[6] Giacomino K, Caliesch R, Sattelmayer KM (2020). The effectiveness of the Peyton's 4-step teaching approach on skill acquisition of procedures in health professions education: a systematic review and meta-analysis with integrated meta-regression. PeerJ.

[7] Gue S, Ray J, Ganti L (2022). Gamification of graduate medical education in an emergency medicine residency program. Int J Emerg Med.

[8] Wang YF, Hsu YF, Fang KT, Kuo LT (2024). Gamification in medical education: identifying and prioritizing key elements through Delphi method. Med Educ Online.

[9] Khamees D, Peterson W, Patricio M (2022). Remote learning developments in postgraduate medical education in response to the COVID-19 pandemic - a BEME systematic review: BEME Guide No. 71. Med Teach.

[10] Krath J, Schürmann L, von Korflesch H (2021). Revealing the theoretical basis of gamification: a systematic review and analysis of theory in research on gamification, serious games and game-based learning. Comput Hum Behav.

[11] Rivera R, Smart J, Sakaria S, Wray A, Wiechmann W, Boysen-Osborn M, Toohey S (2021). Planning engaging, remote, synchronous didactics in the COVID-19 pandemic era. JMIR Med Educ.

[12] Stacey SK, Boswell CL, Cowan KK, Furlano AJ, McEleney MD, Morcomb EF, Tookey CJ (2023). Remote synchronous laceration repair instruction with summary feedback. PRiMER.

